# Using Artificial Intelligence to Establish Chest X-Ray Image Recognition Model to Assist Crucial Diagnosis in Elder Patients With Dyspnea

**DOI:** 10.3389/fmed.2022.893208

**Published:** 2022-06-03

**Authors:** Liu Liong-Rung, Chiu Hung-Wen, Huang Ming-Yuan, Huang Shu-Tien, Tsai Ming-Feng, Chang Chia-Yu, Chang Kuo-Song

**Affiliations:** ^1^Department of Emergency Medicine, Mackay Memorial Hospital, Taipei, Taiwan; ^2^Department of Medicine, Mackay Medical College, New Taipei City, Taiwan; ^3^Graduate Institute of Biomedical Informatics, College of Medical Science and Technology, Taipei Medical University, Taipei, Taiwan; ^4^Clinical Big Data Research Center, Taipei Medical University Hospital, Taipei, Taiwan; ^5^Division of Plastic Surgery, Department of Surgery, Mackay Memorial Hospital, Taipei, Taiwan

**Keywords:** computer-aided detection (CAD), artificial intelligence, geriatrics medicine, critical care medicine, chest X-ray (CXR)

## Abstract

Pneumonia and pulmonary edema are the most common causes of acute respiratory failure in emergency and intensive care. Airway maintenance and heart function preservation are two foundations for resuscitation. Laboratory examinations have been utilized for clinicians to early differentiate pneumonia and pulmonary edema; however, none can provide results as prompt as radiology examinations, such as portable chest X-ray (CXR), which can quickly deliver results without mobilizing patients. However, similar features between pneumonia and pulmonary edema are found in CXR. It remains challenging for Emergency Department (ED) physicians to make immediate decisions as radiologists cannot be on-site all the time and provide support. Thus, Accurate interpretation of images remains challenging in the emergency setting. References have shown that deep convolutional neural networks (CNN) have a high sensitivity in CXR readings. In this retrospective study, we collected the CXR images of patients over 65 hospitalized with pneumonia or pulmonary edema diagnosis between 2016 and 2020. After using the ICD-10 codes to select qualified patient records and removing the duplicated ones, we used keywords to label the image reports found in the electronic medical record (EMR) system. After that, we categorized their CXR images into five categories: positive correlation, negative correlation, no correlation, low correlation, and high correlation. Subcategorization was also performed to better differentiate characteristics. We applied six experiments includes the crop interference and non-interference categories by GoogLeNet and applied three times of validations. In our best model, the F1 scores for pneumonia and pulmonary edema are 0.835 and 0.829, respectively; accuracy rate: 83.2%, Recall rate: 83.2%, positive predictive value: 83.3%, and F1 Score: 0.832. After the validation, the best accuracy rate of our model can reach up to 73%. The model has a high negative predictive value of excluding pulmonary edema, meaning the CXR shows no sign of pulmonary edema. At the time, there was a high positive predictive value in pneumonia. In that way, we could use it as a clinical decision support (CDS) system to rule out pulmonary edema and rule in pneumonia contributing to the critical care of the elderly.

## Introduction

Chest X-ray (CXR) is one of the most commonly used clinical imaging examinations in the medical field due to its adequate image resolution and standardized sampling techniques (1). Before admission to an outpatient clinic or emergency department, patients usually undergo at least one routine CXR, which is rapid and has high diagnostic value for patients displaying symptoms of dyspnea (2). The appearance of pneumonia (PN) on CXR films is inconsistent, and some lung field characteristics, such as infiltration, are similar to pulmonary edema (PE), which is also one of the most severe respiratory diseases. These features were difficult to obtain features with mathematical definitions and traditional image processing methods on CXR. Previous studies suggested CXR performed usually could not be timely interpreted by radiologists to generate proved reports to assist clinicians to make proper diagnosis (3, 4). Even in medical centers of Taiwan, CXR image report generated by radiologist is not as timely as clinical required. Thus, the correct early-stage interpretation of received images is a substantial clinical challenge in emergency and intensive care units.

Although pneumonia and pulmonary edema share some similar characteristics on X-ray films, the main problem in pneumonia is the inflammation of lung parenchyma or interstitium, whereas that in edema is the abnormal accumulation of fluid in the extravascular space of the lung; thus, the pathophysiology and treatment of these diseases are completely different. Pneumonia treatment involves controlling lung infection and relieving inflammation, whereas edema treatment prioritizes the elimination of pulmonary fluid. Appropriate treatment after diagnosis can reduce the duration of hospitalization and may save lives by avoiding respiratory failure; thus, accurately distinguishing these diseases is key for improving patient outcomes (5). In particular, for patients in extreme age groups, namely children and older adults aged 65 years or above, early diagnosis is significantly correlated with mortality rate (6).

AI approach from machine learning to deep learning contributes to comprehensive healthcare in many ways, such as: symptoms detection, disease classification. Not only has the opportunity to improve the diagnosis and helping decision-making, but also has the potential reduce the cost of medical care (7). Deep learning can be used to identify and derive meaning from image features. Its performance in image recognition tasks has been confirmed in previous studies; deep learning has performance superior to conventional machine learning in the medical filed (8), and can be used in computer-aided detection (CAD) (9). Recently, deep learning has been applied for clinical decision-making assistance for the diagnosis of various diseases, because of it is efficient to deal with unstructured and ambiguous data (10), including diabetic retinopathy, macular edema (11, 12), skin cancer (13), and breast cancer (14). CXR is one of the most commonly used examinations in hospitals, and numerous CXR images can be easily obtained. However, laboratory findings are always more trustworthy than diagnosis based on image features alone, which often challenge early diagnosis. Deep learning models would helped in recognize complex patterns precisely (15). Many papers have used deep learning to help identified chest lesions such as pneumonia, pneumothorax, etc. (16, 17), Furthermore, the specific pattern of pneumonia caused by Covid-19 could also be recognized by deep learning method (18). Deep convolutional neural networks (CNNs) have exceptional performance in image classification. In 2012, CNNs demonstrated excellent image recognition performance in the ImageNet Large-Scale Visual Recognition Competition (ILSVRC) classification task challenge (19). CNNs have a multilayer neural network structure with strong fault tolerance, self-learning, and parallel processing capabilities. In CNN learning, suitable features can be selected as inputs without additional manual processing, the features can be automatically analyzed from the original image data, and feature classification can be learned. CNNs use convolutional layers to extract features and use pooling (max or average) layers to generalize features. The set of the various filters they used for Convolutional Layers extract different sets of features. The biggest advantage of Deep Learning is that we do not need to manually extract features from the image. The network learns to extract features while training. Thus, CNN learning considerably reduces manual preprocessing, facilitating the learning and classification of optimal visual features. Compared with the general feedforward network, the local connection method of the CNN greatly reduces the network parameters. Many CNNs have been developed, such as AlexNet (20), GoogLeNet (21), ResNet (22), and VGGNet (23).

Numerous studies have verified that CNNs for lung disease identification can produce diagnosis results with accuracy meeting that of radiologists, such as ChestX-ray14, which is used public datasets of National Institutes of Health (24), and the CheXNeXt, which is based on the DenseNet (25). However, research for critical cases or cases in older adults were not mentioned in previous studies. The standard CXR uses the posterior-anterior view (PA view) and is performed with the patient standing. The PA view is optimal for image interpretation and for analysis of the mediastinal space and lungs and can be used for accurate heart size assessment (26). For patients with severe illness who are bedridden or unable to stand, the anterior-posterior view (AP view) or portable CXR are alternate methods. Because the heart is located further away from the film, the AP view may cause the ratio of the heart to the mediastinal space to be enlarged by 15–20%, affecting the clinician's judgment of the sizes of the heart, blood vessels, and lymph vessels in the anterior mediastinal space. Moreover, factors such as enlarged mediastinal space, elevated diaphragm, skin folds, and incomplete opening of the scapula in the AP view can affect the physician's interpretation and increase the possibility of errors (27). Furthermore, patients with critical illnesses are often have life support instruments such as endotracheal tube or vitals monitoring equipments attached to their body likes electrocardiograph wires. Given CNNs' high potential for identifying tiny particles or objects in images (28), studies have not individually discussed the interference caused by these instruments or have even excluded this group of patients. Although these images are the most challenging for machine learning, the capacity for interpreting them in clinical practice is urgently needed.

In this retrospective study, we discussed approaches of distinguishing between pneumonia and pulmonary edema on radiograph. GoogleNet transfer learning was used to analyze the performance of machine learning in distinguishing between PN and PE in the chest radiograph of patients aged 65 years or older who were admitted to the emergency department in Mackay Memorial Hospital. Moreover, we explored the effects of instrument interference, image cropping, and text labels on the capacity of machine learning to classify images. The objective of this study was to establish a CAD model for early diagnosis aimed at patients with critical illnesses.

The main contributions of this study are as follows:

- We provide CAD tools for critically ill elderly who urgently need assistance in image interpretation.
- We demonstrate the interferences such as life-supporting catheters? instruments affect the machine learning outcomes.
- The performance of machine learning in chest X-ray is consistent with the radiologists, when the EMR have more clear features such as pneumonia and edema, the better results are trained on these images.

## Materials and Methods

**Figure 1:**
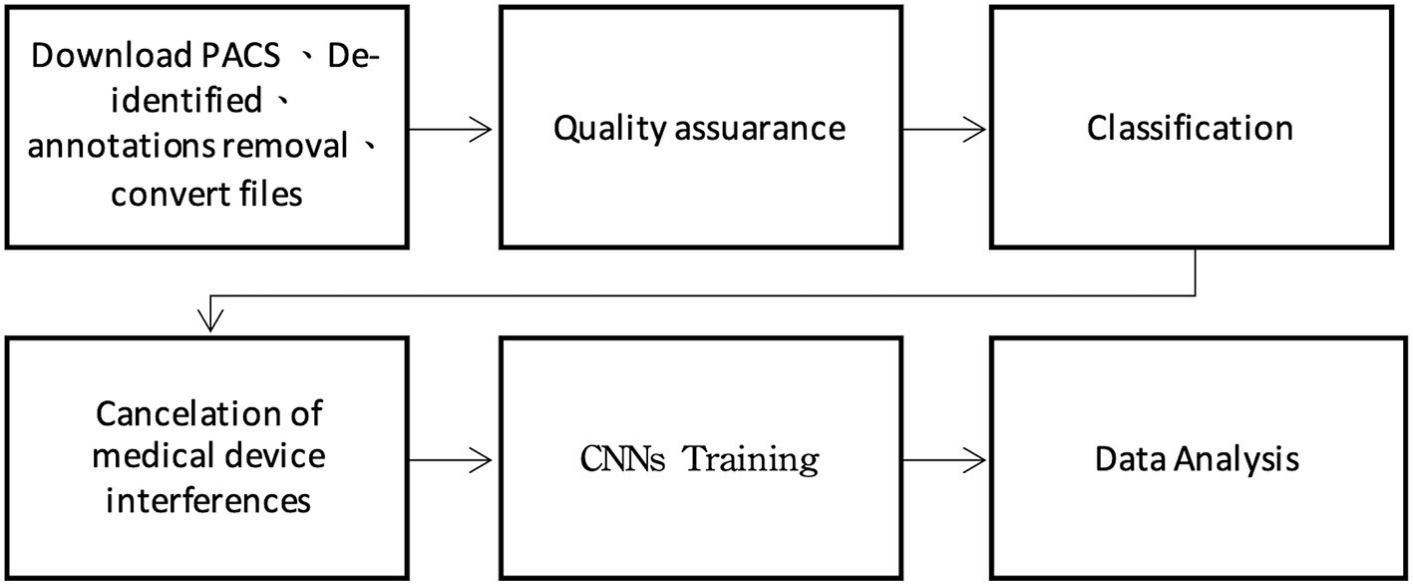
Data processing procedure.

This study was approved by the Institutional Review Board of Mackay Memorial Hospital. The *International Classification of Disease, Tenth Revision* (ICD-10) hospital discharge codes collected in one medical center in Taiwan (Mackay Memorial Hospital) since 2015 to 2020 for patients aged 65 years and older who were admitted to the hospital through the emergency department. The number of CXR images from patients with PN (ICD-10: J18) and PE (ICD-10: J81) were 45,781 and 43,674, respectively. Moreover, the electronic medical records (EMR) compiled by radiologists were labeled using keywords and subsequently analyzed by two emergency physicians with more than 15 years of experience. A plastic surgeon assisted with image classification and training. The experiment was divided into six steps comprising tasks including preprocessing, text labeling, and machine learning (Figure 1).

### Data Acquisition

The CXR images were downloaded through the picture archiving and communication system (PACS), after which deidentification and annotation removal were performed. Moreover, 800 CXR images of patients without lung disease at admission were collected and similarly subjected to deidentification and annotation removal for joint training of the proposed CAD model with the CXR images. The training image format was JPG, and the image conversion size was 224 × 224 × 3 pixels.

### Quality Assurance

**Figure 2:**
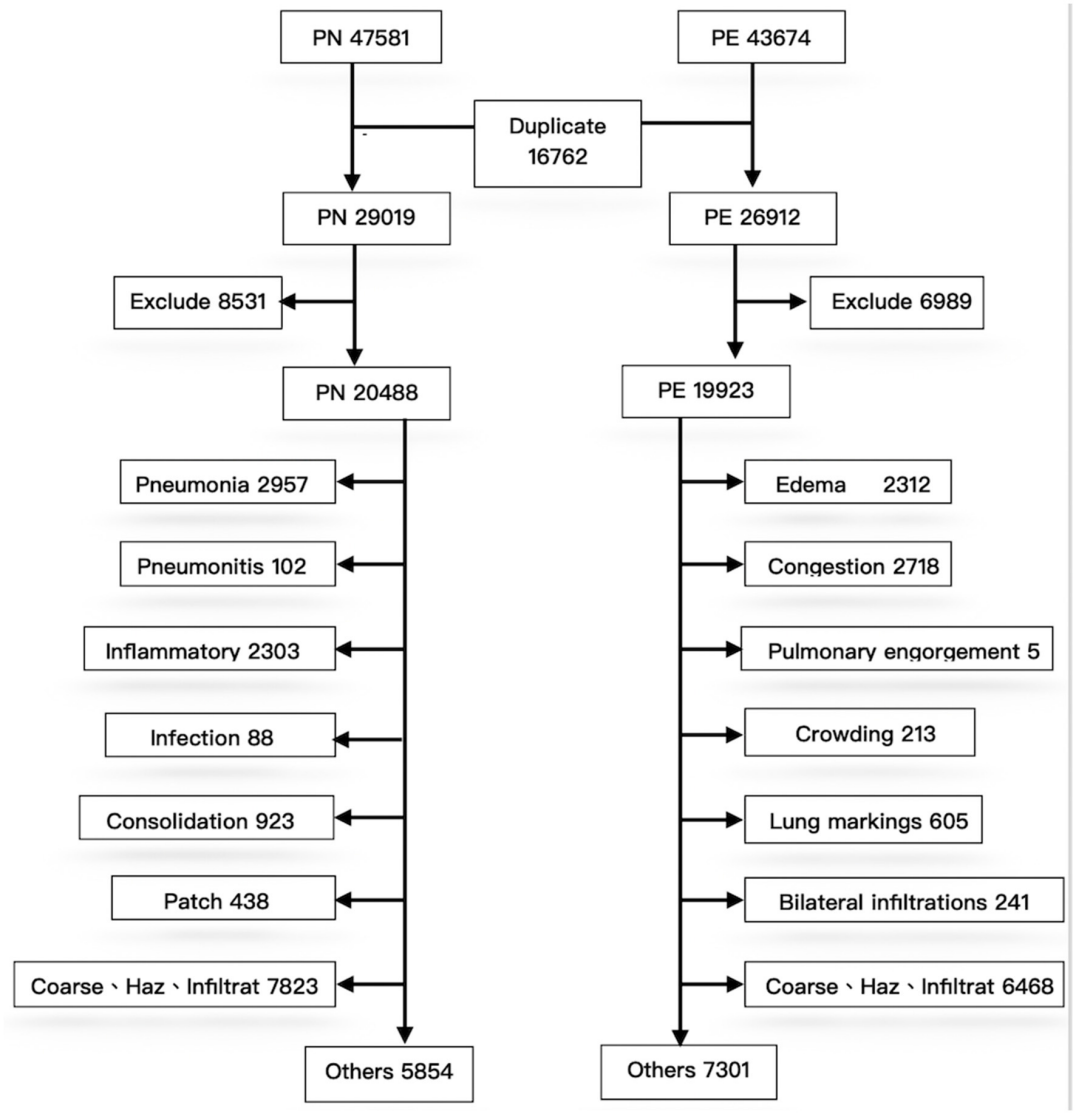
Classification process for image data. PN, pneumonia; PE, pulmonary edema.

To exclude repeated cases and ensure the quality of machine learning, a pretraining process using image numbers and text labels was performed before training the CNN (Figure 2).

First, during deidentification, we discovered that data were repeated in the PN and PE sets; 16,762 images were present in the two disease lists, indicating that both J18 and J81 were included in the ICD-10 codes of these cases. These duplicated images were not errors in case collection; instead, they represented older adults with multiple comorbidities. For example, many patients with severe pulmonary edema (e.g., acute decompensated heart failure) were complicated by pneumonia due to respiratory tract infection after hospitalization. Conversely, patients with pneumonia may also develop multiple organ failure after hospitalization, leading to edema (e.g., heart or renal failure). Therefore, duplicated images of the two diseases are expected and reasonable in the collection of clinical cases. Accordingly, the 16,272 repeated cases were excluded; otherwise, they could not be classified during CNN training. After exclusion of the repeated cases, PN and PE each had 29,019 and 26,912 images. Because the data were obtained directly from the PACS system, some erroneous data might be included. After reconducting a query of reports using keywords to exclude irrelevant cases, the PN and PE data sets had 20,488 and 19,923 images, respectively.

### Experimental Design for Image Classification

The effects of CNN on the interpretation results under different conditions were investigated with six experiments as follows.

Experiment 1: First, we tested the CNN's capability to identify diseases and its capacity to distinguish between PN and PE with correct ICD-10 diagnoses. A total of 2,000 files were randomly sampled from the 20,488 PN and 19,923 PE images and were combined with the 800 images of patients without lung disease for transfer learning. The training model was named G_random.

Experiment 2: Because the images collected in this study were those of patients with critical illnesses, more than half of the images contained extracorporeal life support instruments or tubes. To determine the degree of interference of this equipment on machine learning, the 2,000 PN and PE images were further divided into images with and without interference; these sets were independently used to train the machine learning model. For images with interference, we randomly sampled 1,000 files from the two disease data sets for training. This training model was named G_int.

Experiment 3: PN and PE were confirmed to contain only 650 and 480 images without interference, respectively. We named this training model G_NCC and determined whether superior results were obtained for the images without interference.

**Figure 3:**
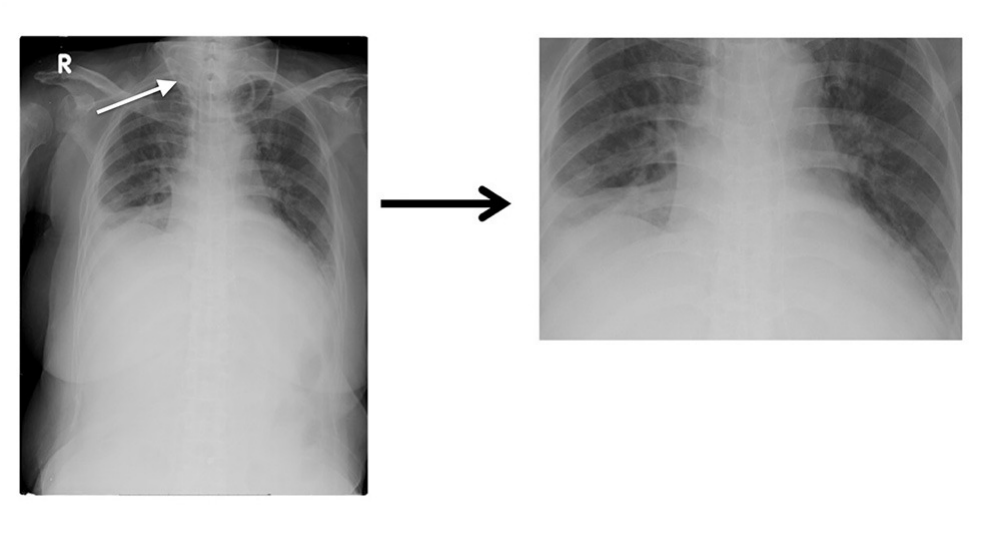
Comparison of images before and after manual cropping.

Experiment 4: To improve the training model, the images with interference were processed using image cropping. In Figure 3, the oxygen supply mask (indicated by a white arrow in the image) was cropped to produce a clearer lung field. Finally, we processed 1,100 PN and 670 PE images; this training model was named G_clean.

Experiment 5: To further determine whether EMR labels precisely would produce superior results in training, 2,000 each image which obviously clamed pneumonia and edema were collected and separated into two categories; this model was named G_DC2.

Experiment 6: The data from Experiment 5 were combined with 800 normal CXR images for joint training; this model was called G_DC. Experiments 5 and 6 were performed to compare whether machine learning was affected by including the comparatively easily identifiable normal CXR images.

### Model Training

The built-in neural network toolbox of MATLAB R2020b (The MathWorks, Natick, MA, USA) on Windows 10 (Microsoft, Redmont, WA, USA) was used for the experiments. The computer had a GeForce RTX 2060 (Nvidia, Santa Clara, CA, USA) graphics processing unit, and the training image format was 24-bit JPG.

The transfer learning used the GoogLeNet Inception V4 architecture. GoogLeNet is a type of convolutional neural network based on the Inception architecture (29). It utilizes Inception modules, which allow the network to choose between multiple convolutional filter sizes in each block. An Inception network stacks these modules on top of each other, with occasional max-pooling layers with stride 2 to halve the resolution of the grid. The GoogLeNet we used in this study 22 layers deep and have an image input size of 224-by-224.The data were trained through multilayer calculations, and the composition of each layer was automatically learned from the data set. A key feature was the Inception module, which was regarded as a milestone in the history of CNN development in a previous study (30). Because this module replaces the fully connected structure with sparse connections for the input images, performs multiple convolution operations or pooling operations, and splices all of the output results into an extremely deep feature map, the module reduces the computational burden of including numerous parameters as well as the problem of overfitting. The performance of the current iteration of Inception, Inception-v4, was verified in the 2015 ILSVRC challenge; it has superior image recognition capabilities due to its use of residual Inception networks.The training environment settings were as follows: minimal batch size = 20, maximum epochs = 50, pixel range= [−3, 3], Rotation Range = [−15, 15], and training/validation ratio = 70:30.

### Model Performance Evaluation

The built-in neural network toolbox in MATLAB R2020b was used to draw the receiving operating characteristic (ROC) curve and produce a confusion matrix. The recall, precision, F1 score, and accuracy of each model were then calculated. Recall, precision, and F1 Score are frequently used for analyzing model performance. A high F1 score indicates higher precision and recall for disease decision-making, and the results of the aforementioned transfer learning models were analyzed using these indicators.

## Results

Table 1 presents the model performance evaluation results for all six experiments. The G_DC model that used images clearly identified as having PN or PE had the highest accuracy and F1 score. The F1 score, and accuracy of the G_DC model (F1 score = 0.882, validation accuracy = 86.4%) were significantly superior to those of the G_random model that was trained using only ICD-10 codes (F1 score = 0.82; validation accuracy = 79.1%).

**Table 1:** Accuracy, recall, precision, and F1 score results for the six experiments.

	**Recall**	**Precision**	**Accuracy**	**F1 score**
G_random	81.3%	82.8%	79.1%	0.82
G_int	73.2%	73.4%	73.4%	0.733
G_clean	73.5%	77.9%	77.5%	0.756
G_NCC	74%	73.6%	74.1%	0.738
G_DC	87.7%	88.7%	86.4%	0.882
G_DC2	83.2%	83.3%	83.2%	0.832

*G_random: Randomly selected from the PN and PE category and combined with normal CXR.

*G_int: Images with interferences from G_random*.

*G_clean: Images with interferences form G_random cropped manually*.

*G_NCC: Images without interferences from G_random*.

*G_DC: G_DC2 combined with 800 normal CXR images*.

*G_DC2: Images labeled pneumonia and edema*.

In addition, the G_int model that was trained solely using images with interference had the worst results with an accuracy of only 73%. Both the G_NCC model, which trained on images without interference from the beginning, and the G_clean model, which trained on cropped images, did not have significant improvements in their validation accuracy or F1 scores. In addition, the G_clean model had a significantly increase for recall of PN from 78.3 to 90.5%; however, its PE recall declined from 79.2 to 56.4%. No significant change was observed in the precision for the two diseases (PN: 76.7 to 77.1%; PE: 77.4 to 78.6%).

**Figure 4:**
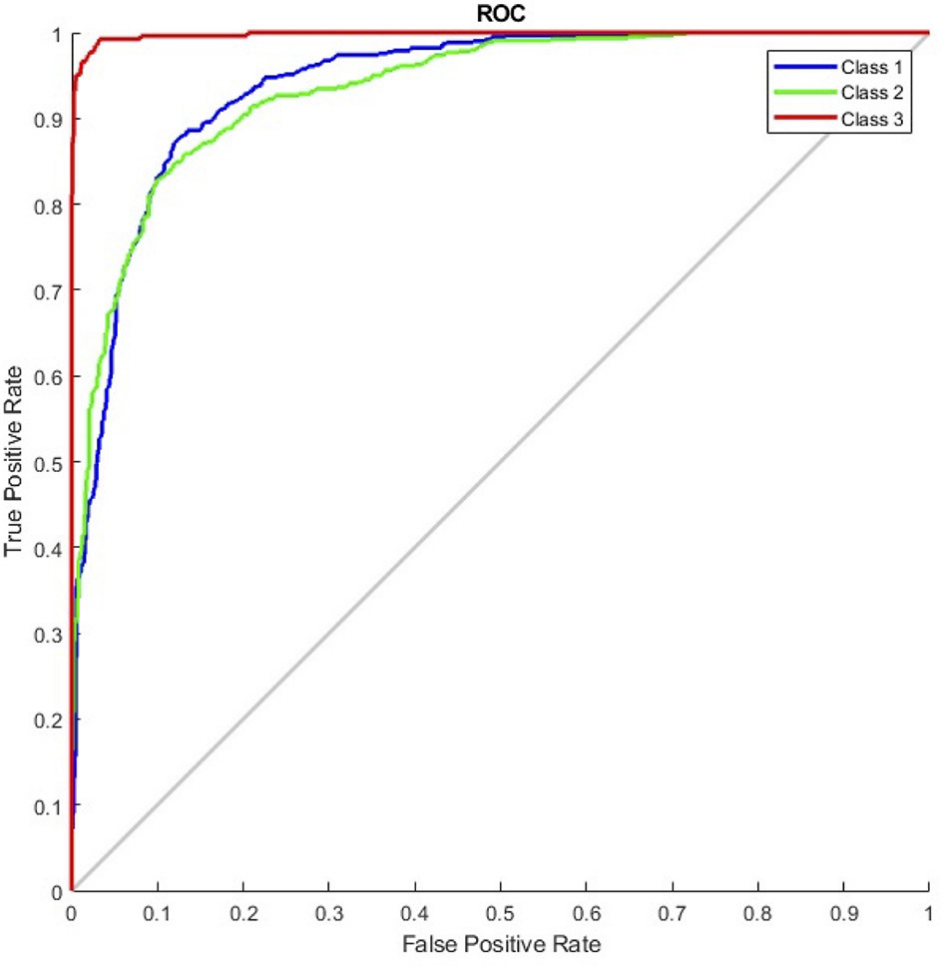
Receiver operating characteristic curve of the G_random model (class 1, pulmonary edema; class 2, pneumonia; class 3, normal).

**Figure 5:**
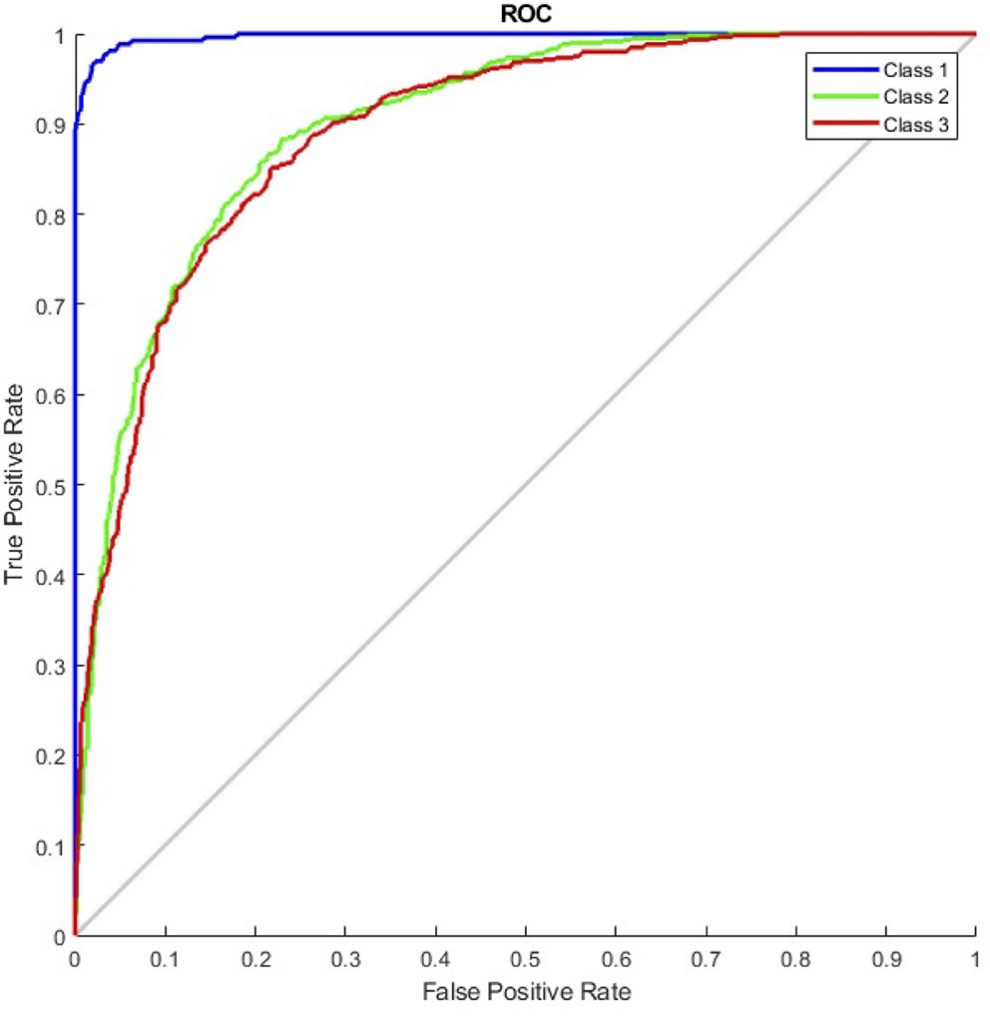
Receiver operating characteristic curve of the G_DC model (class 1, normal; class 2, pulmonary edema; class 3, pneumonia).

The results of the G_random and G_DC models, the training of which incorporated normal CXR images, revealed that normal CXR resulted in an optimal area under the ROC (AUC; Figures 4, 5), indicating that normal CXR images are easier to identify.

Based on the aforementioned results, we believed that, rather than medical interferences, images used for training with more precise description from EMR were the decisive reason that affected machine learning performance; such images proved to be the main factor for improving machine learning performance.

## Discussion

### Effects of Incorporating Normal CXR

In the experiments, normal CXR images of patients without PN or PE had an F1 score over 95%. Machine learning had superior performance among normal CXR than radiographs with lesions. Similar results also demonstrated by Cicero et al. (21), the positive predictive value of the normal category reached 90%, whereas those for the consolidation and for edema were merely 23 and 43%, respectively. Thus, training with normal CXR images could raise overall model accuracy by increasing both the true positive and true negative values.

### Comorbidities

For cases that might be diagnosed with PN and PE simultaneously, we used two steps for preprocessing: (1) excluding 16,762 repeated files on ICD-10 diagnosis; and (2) excluding images based on the imaging reports. In machine learning, feature selection is considered a critical step in data preprocessing. When we directly use raw data such as ICD code for classification, we sometimes observe that learning algorithms perform poorly (31). These images were excluded because our experiments did not aim to identify comorbidities and the presence of two or more diseases in one radiograph would reduce the machine learning performance (32).

### Interferences

There is a significant difference influence between machine learning and physician interpretation for medical devices and life support equipment. Those *in vitro* instruments do not affect physicians reading images, whereas machine learning can detect even subtle features that would not normally be detected (33), affecting the learning outcome. In our study, the G_int model that all images with medical equipment for training had a significant decrease in its predictive performance; its accuracy was reduced from 79.1 to 73.4%, and its F1 score was reduced from 0.82 to 0.733 (Table 1). We performed image processing using cropping but did not obtain a more favorable result. As we known, machine learning is more efficient in distinction of localized lesions rather than lesions with global symmetrical patterns (25). Therefore, pneumonia which sometimes shows unilateral consolidation is easier to be identified than pulmonary edema which is bilateral symmetrical pictures.

The cropped images differed substantially from the original images; this may explain why the training performance was worse than as expected. Moreover, the ratio of the lung field to the lesions might have changed after cropping, causing local consolidation, which were originally easily identified by the models, to exhibit features that more closely resembled diffusion. In addition, for patients in critical condition, PE images almost always contained one or more medical instruments or life support tubes, leading to the exclusion of many images that could not be fully cropped in training. Only 1,100 and 670 PN and PE training images, respectively, were retained after cropping. Moreover, we discovered that the recall of PN was significantly higher than that of PE (90.5 vs. 56.4%). The number of images in training sets must be balanced because an unbalanced number of training images causes learning to be biased toward image types that the model had more exposure to (34). Thus, insufficient datasets and unbalanced training sets might also have affected the performance.

### Model Comparison

There were many previous studies used CNN as a chest X-ray CAD tools. Some models were published based on public institutions datasets such as ChestX-ray14 which built by The National Institutes of Health (35). Cicero et al. used GoogLeNet in 2017 to construct a model that resolves a total of about 35,000 images. It includes normal chest plain films and other five features: Pleural effusion, Cardiomegaly, Consolidation, Pulmonary edema and Pneumothorax. It is found that normal chest plain films had the best recognition, which both sensitivity and specificity can reach above 91%. CheXNeXt used ChestX-ray14 datasets compared with radiologists for identification of 14 chest X-ray features in 2018. Results showed that CheXNeXt performed as well as radiologists on 10 features (no statistically significant difference in AUC) and it was superior than expert on atelectasis. Not as good as radiologists on three characteristics (cardiomegaly, emphysema, emphysema). We compared the performance of pneumonia and pulmonary edema in G_DC and G_DC2 with above literature models. In our experiments both pneumonia and pulmonary edema have higher sensitivity, PPV and F1 score (Table 2).

**Table 2:** Experiments 5 and Experiments 6 compares with previous study.

	**Cicero et al. (21)**	**CheXNeXt**	**G_DC**	**G_DC2**
PE sensitivity	0.82	0.682	0.868	0.834
PE PPV	0.43	0.662	0.83	0.825
PE F1 score	0.564	0.672	0.849	0.829
PN sensitivity	0.74	0.650	0.832	0.83
PN PPV	0.23	0.377	0.852	0.84
PN F1 score	0.351	0.477	0.842	0.835

### Limitations

It has been demonstrated that medical history and laboratory tests would improve radiologist interpretations (36). In this study, we did not combine patients' history and clinical data together for thorough analysis which might provide important part in clinical CAD tool. In addition, due to the limitations of deep learning, our tools currently cannot articulate the eigenvalues by which to classify images. Data preprocessing and text labeling both revealed that PN and PE are related to many diseases and share mutual comorbidities. To maintain a simple training environment during data processing, cases with shared comorbidities were excluded, and no further analysis was conducted on the interpretation of comorbidities. The data in our study were collected from a single medical center, which might affect the objectivity of the text labels. Finally, we did not test the models against the interpretation of the radiologists; thus, we were unable to compare the similarities and differences between the interpretation of the models and specialists.

## Conclusion

This study revealed that using deep learning to construct X-ray images and to distinguish between PE and PN, and using images with explicit signs of PE or PN and without interference for training, can produce an accuracy of over 80%. Moreover, an accuracy of 70% or higher was achieved even in the presence of interference. In addition, the recognition rate of normal images exceeded 90%; thus, this model can be potentially applied in clinical practice.

Currently, more than two-thirds of the world's population do not have access to professional interpretation of medical images, are unable to receive timely diagnosis reports, or cannot receive any diagnosis. During emergencies or the presence of large number of patients in medical centers (e.g., COVID-19 outbreak clusters), experienced radiologists are subject to human limitations, such as off duty hours, fatigue, and perceptual and cognitive biases; these limitations may lead to misjudgment. Although our model cannot completely replace clinicians. After testing, our model showed excellent performance on identifying pulmonary edema and also informative assistance on patients with pneumonia in elder patients after testing. It provides crucial image information in a timely manner to assist in clinical diagnosis.

## Data Availability Statement

The original contributions presented in the study are included in the article/supplementary material, further inquiries can be directed to the corresponding author/s.

## Ethics Statement

The studies involving human participants were reviewed and approved by Mackay Memorial Hospital Institutional Review Board (IRB). Written informed consent for participation was not required for this study in accordance with the national legislation and the institutional requirements.

## Author Contributions

LL-R conducted experiments and wrote the manuscript. HM-Y built chest X-ray datasets. HS-T, TM-F, and CC-Y helped selecting patients and organized electric medical records and data assembling. CK-S provides administrative support. CH-W developed the methods, contributed to manuscript editing, and reified experiment instructions. All authors contributed to the article and approved the submitted version.

## Conflict of Interest

The authors declare that the research was conducted in the absence of any commercial or financial relationships that could be construed as a potential conflict of interest.

## Publisher's Note

All claims expressed in this article are solely those of the authors and do not necessarily represent those of their affiliated organizations, or those of the publisher, the editors and the reviewers. Any product that may be evaluated in this article, or claim that may be made by its manufacturer, is not guaranteed or endorsed by the publisher.
